# Experimental data from the development of *Lymnaea stagnalis* embryo test for chemicals hazard assessment

**DOI:** 10.1016/j.dib.2024.110324

**Published:** 2024-03-12

**Authors:** Ricardo Capela, Luís Filipe Castro, Miguel Machado Santos, Jeanne Garric

**Affiliations:** a CIMAR/CIIMAR – Interdisciplinary Centre for Marine and Environmental Research, Av. General Norton de Matos s/n, 4450-208 Matosinhos, Portugal; b FCUP – Faculty of Sciences of the University of Porto, Rua do Campo Alegre s/n, 4169-007 Porto, Portugal; c INRAE - National Research Institute for Agriculture, Food and the Environment - Centre de Lyon-Villeurbanne, 5 rue de la Doua, CS20244, 69625 Villeurbanne Cedex, Lyon-Villeurbanne, France

**Keywords:** Embryo toxicity, Mollusca, Model species, Risk assessment, Bioassay, Biodata, Cadmium

## Abstract

This study aimed to contribute to the development of an embryo-test using the gastropod *Lymnaea stagnalis*, identified by the Organization for Economic Co-operation and Development (OECD) as a potential invertebrate test animal model. Together with the *Potamopyrgus antipodarum*, were the first mollusc models to be included in the organization testing guidelines. The focus was on validating an embryo toxicity test to cover the sensitive embryogenesis phase and on obtaining testing information on all of the model life cycle stages, contributing to close an identified gap within this context. Adhering to OECD guidelines, namely the L. *stagnalis* reproductive test, the study examined mortality rates, abnormality rates, development, growth, hatching rates, hearth rates, and pre-testing media suitability, during the embryogenesis, and the obtained dataset made available for further studies. Cadmium was chosen as the positive test compound due to its well-studied nature and the model's proven sensitivity to the compound, working as a reference compound for the test development.

The data were collected in two 12-day assays under consistent conditions, each using 144 L. *stagnalis* embryos (<24 h old) from 6 egg masses (288 embryos total). Six 48-well microplates were utilized per assay, accommodating five different cadmium concentrations (32, 70, 155, 341, 750 µg/L) and a control group.

Recorded parameters encompassed developmental stage, embryo position within the chorion, developmental abnormalities, hatchings, and mortality. Data analysis involved classifying embryos based on developmental stage and position, taking an exploratory approach to define the relevance of the different parameters in the compound hazard assessment during the embryogenesis. Measurements considered embryo area, perimeter, length, height, width, interocular distance, and heart rate.

This dataset does not provide treated information but the raw data obtained during the proposed metodological development and toxicity testing process. The purpose of this article is to make the obtained raw data available, clearly defining the acquisition methodology to provide a comparison basis for future or existent works within this context.

Specifications Table

**Table:** 

Subject	Health, Toxicology and Mutagenesis
Specific subject area	The work focus in the field of the Ecotoxicology, particularly hazard and Environmental Risk Assessment (ERA). The study addresses the development of an embryo tests with a freshwater gastropod for hazard assessment of chemicals and contaminants in the aquatic ecosystems.
Data format	Raw
Type of data	Table
Data collection	Data collection involved two 12-day assays under consistent conditions, each using 144 L. *stagnalis* embryos (<24 h old) from 6 egg masses (288 embryos total). Six 48-well microplates were utilized per assay, accommodating different cadmium concentrations (32, 70, 155, 341, 750 µg/L) and a control group. Parameters such as pH, dissolved oxygen, conductivity, mortality, developmental stage changes, abnormalities, and growth were monitored. Heart rate was recorded at 168 h, and hatchings were documented (Table 1). Data acquisition included stereomicroscope observations (Nikon SMZ) and photography (Leica MC170 HD camera with Leica Applications Suite – LAS v4.5). Measurements were obtained with ImageJ v.1.52a, Wayne Rasband, NIH, USA (Figure 1). Recorded parameters encompassed developmental stage (Figure 2), embryo position within the chorion, developmental abnormalities, hatchings, and mortality. Data analysis involved classifying embryos based on developmental stage and position, with exploratory approaches to define relevant parameters. Measurements considered embryo area, perimeter, length, height, width, interocular distance, and heart rate (Table 2).
Data source location	These data were collected under laboratorial conditions in the INRAE - National Research Institute for Agriculture, Food and the Environment - Centre de Lyon-Villeurbanne; 5 rue de la Doua, CS20244, 69,625 Villeurbanne Cedex, Lyon-Villeurbanne, France. They are currently stored in the CIMAR/CIIMAR – Interdisciplinary Centre for Marine and Environmental Research, *Av*. General Norton de Matos s/n, 4450–208 Matosinhos, Portugal.
Data accessibility	Repository name: Mendley data Data identification number: 10.17632/f7p8vwwh7y.1 Direct URL to data: https://data.mendeley.com/datasets/f7p8vwwh7y/1 Capela, Ricardo; Castro, Luis Filipe; Santos, Miguel; Garric, Jeanne (2023), “Lymnaea stagnalis embryotest datafile”, Mendeley Data, V1, doi: 10.17632/f7p8vwwh7y.1
Related research article	Development of a *Lymnaea stagnalis* embryo bioassay for chemicals hazard assessment. [1]

## 1 Value of the Data

• Standardization Support: These data lay a robust foundation for future standardization of invertebrate-based toxicity tests, fostering consistency and reliability in hazard assessment practices.
• These data provide valuable insights into the sensitivity of L. stagnalis embryogenesis to cadmium, enhancing our understanding of toxicological effects during this critical developmental stage.
• The availability of complete datasets regarding the optimization and validation of new testing methodologies may accelerate development of these methodologies, providing valuable data for training and results validation.
• This dataset may be of relevant importance to scientists or even entities working on the development, validation and standardization of test guideline protocols.
• The dataset supports the 3R policy by offering an alternative to traditional vertebrate and arthropod testing, reducing the need for animal experimentation, and aligning with best ethical research practices.

## 2 Data Description

The dataset is composed of a single excel file containing all the relevant data acquired during the development and optimization of an embryo-test using the freshwater gastropod *Lymnaea stagnalis*. The file is composed by three sheets (Captions, Incubation media assay and Cadmium assays). The first sheet (Captions) contains the captions to guide the user trough the file. The second sheet (Incubation media assay) contains the acquired data from a first pre-incubation of two incubation media, which was carried out during 168 h and the measurements taken every 24 h; Length (µm), the embryos length in the two main axis (L1 – ‘length’, larger axis, and L2 – ‘width’, smaller axis), Area (µm²) and Perimeter (µm) are provided for each measurement point. The third sheet (Cadmium assays) contains the complete set of data acquired during the exposure of the embryos to the reference compound, cadmium. It contains data regarding the experimental design, developmental events (passage of stages and malformations during the developmental process), survival/mortality and biometric measurements. The first six collumns (A to F) identify the assay design: Experiment, two independent assays were carried out (1 and 2); the Plate (microplate numeration for each tested condition); Plate Well – row and column, identifies each sample location within the plate; Group, refers to each exposure group: Dose, compound concentration (cadmium) for each group in micrograms per liter (µg/L). The following collumns (collumns H to BV) present the acquired information during the assay in each measurement time-point: Exposure time (in hours); Position, embryo position in the chorion when the measurement was taken; Development, the embryo developmental stage at the measurement point; Malformations, characterizing the embryo development (normal, abnormal - malformation type and mortality); Length (L1 – larger axis, and L2 – ‘width’, smaller axis), Area (µm²) – embryo area; Perimeter (µm), the embryo perimeter. After the 96 h of exposure the Interocular distance was also measured – IOD (µm). Collumn AY - Hearth beatings (beats/20 s), the hearth beatings were measured at the 168 h, in 20 second intervals. After the 192 h, only the embryo development was recorded (Development and Malformations).

## 3 Experimental Design, Materials and Methods

### 3.1 Experimental design

Two identical 12-day assays were conducted under the same conditions. Each assay featured 144 L. *stagnalis* embryos, all under 24 h old, originating from 6 separate egg masses, totaling 288 embryos from 12 egg masses.

For each assay, six 48-well microplates, each containing 1 mL of working solution, were employed. Every plate was dedicated to testing a specific cadmium concentration (32, 70, 155, 341, and 750 µg/L of Cadmium), with one plate designated for the control group. Cadmium was chosen as the positive test compound due to its well-studied nature in Biosystems and environment [2], [3], [4] and the model's proven sensitivity to the compound. It was also used as reference in the *Lyamnea stagnalis* OECD reproductive test guideline working as a reference compound for the test development, providing the basis to define the models sensitivity in the current testing conditions when compared to the available test guidelines and models [1,[5], [6], [7]].

To ensure precision, only one cadmium concentration was assessed per plate. Each plate included 4 internal control embryos and 20 exposed animals, in line with OECD guidelines [7,8] specifying a spacing factor of 2.2 fold between concentrations.

Throughout the assays, critical physical-chemical parameters (pH, dissolved oxygen, and conductivity) were documented before and after medium renewal on days 2, 4, 6, 8, and 10.

On a daily basis, various endpoints such as mortality, developmental stage changes (Fig. 2), and abnormalities were examined. Animals were regularly photographed to estimate growth. Furthermore, heart rate measurements were recorded at 168 h, hatchings were registered, and the animals were weighed to provide comprehensive data on their development and response to cadmium exposure.

### 3.2 Data acquisition methods

**Fig. 1:**
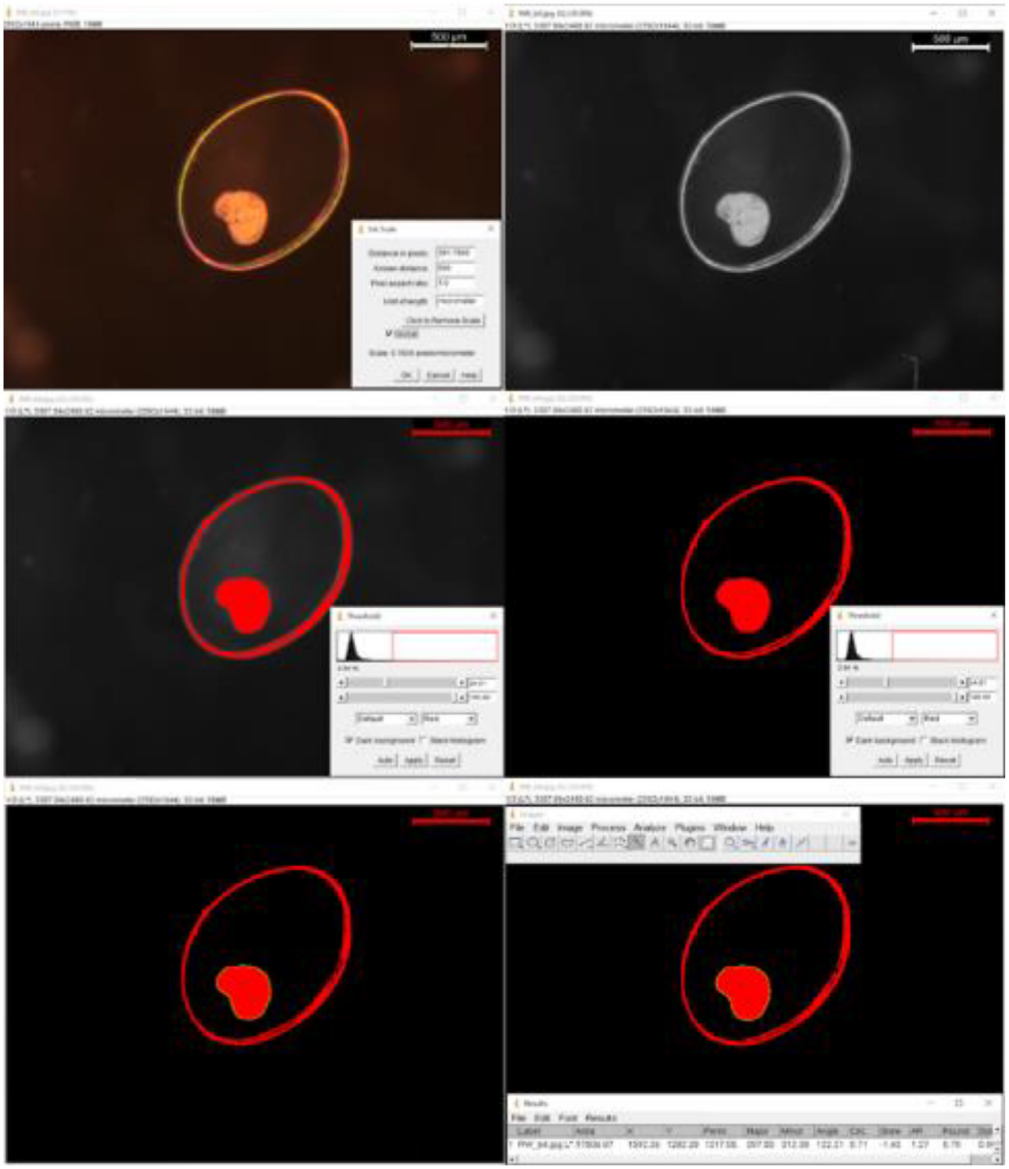
ImageJ measurement sequence; Image – Incubation media assay, RW (reconstituted media) day 3; RW_b4; a) Set Scale; b) Image > Type > Lab Stack (32 bit); c) Image > Ajust > Threshold; d) Set image treshold parameters; e) Wand tool (outline the interest section); f) Measure (perimeter – outline perimetr measure; area – outlined area, red area [1].

Embryo observations occurred at 24 ± 1 hour intervals under a Nikon SMZ stereomicroscope. Simultaneously, Leica MC170 HD camera equipped with Leica Applications Suite – LAS v4.5 was employed for photographic documentation. Measurements were conducted using ImageJ v.1.52a, a software developed by Wayne Rasband at the National Institutes of Health in the USA (Fig. 1).

**Fig. 2:**
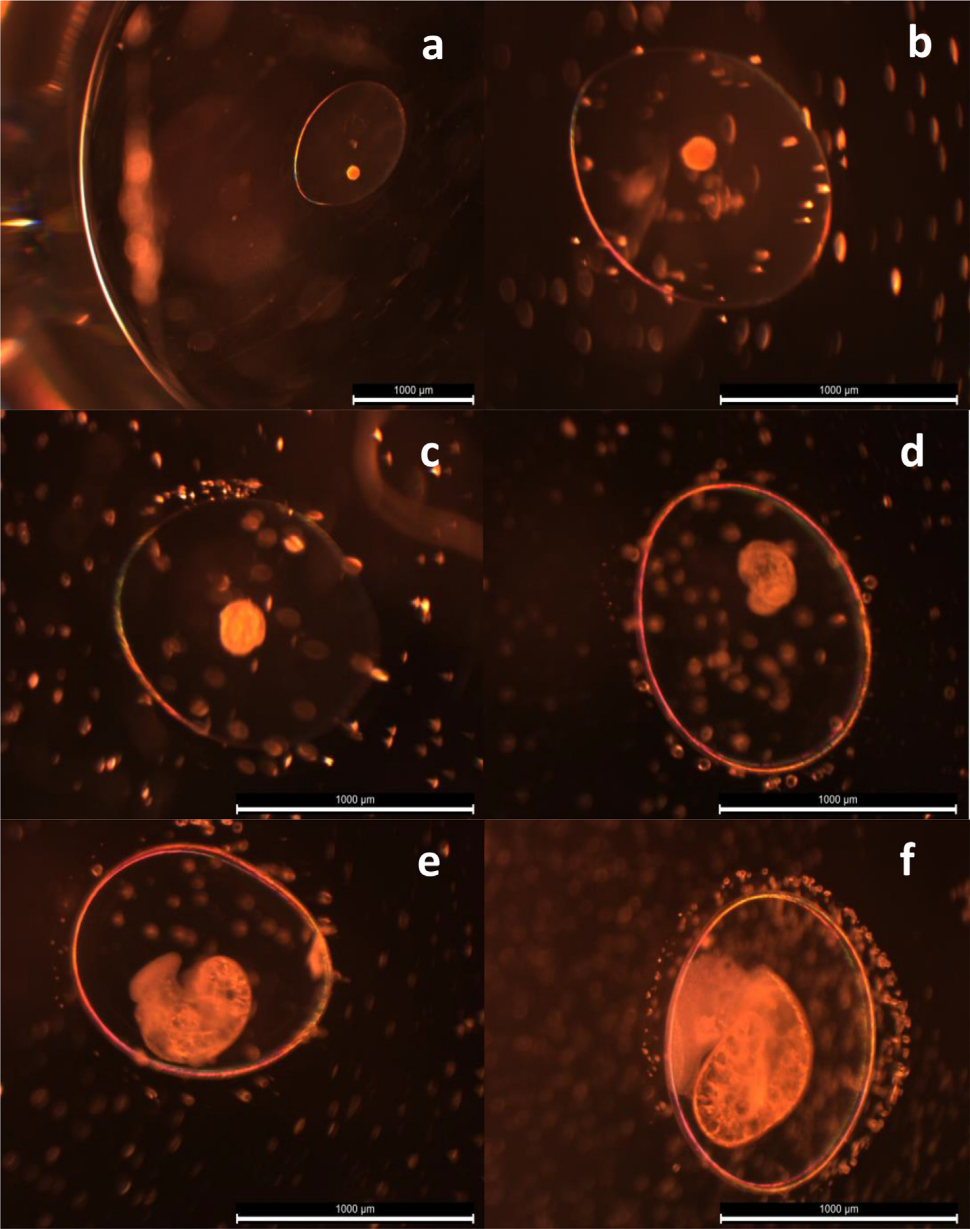
*Lymnaea stagnalis* developmental stages. a) morula; b) Early trochophore; c) Mid trochophore; d) Late trochophore; e) Velliger; f) Hippo stage.

Throughout the test duration, a comprehensive set of parameters was recorded for all embryos. These parameters included developmental stage assessment, embryo positioning within the chorion, detection of developmental abnormalities, tracking of hatchings, and monitoring of mortality (Tables 1 and 2).

**Table 1:** *Lymnaea stagnalis* 12 days cadmium embryo test assay characterization.

Plate	Plate Well Row	Plate Well Column	Group	Dose (µg/L Cd)	Exposure time (h)
One plate per treatment: Control plus exposure groups (with internal control - iC)	48 Well microplates: rows B to E	48 well microplates: columns 2 to 7	Control	0	12 days assays: time-points each 24 h (48, 72, 96, 120, 144, 168, 192, 216, 240, 264 and 288 h)
			iC	0	
			32 µg/L	32	
			70 µg/L	70	
			155 µg/L	155	
			341 µg/L	341	
			750 µg/L	750

**Table 2:** *Lymanea stagnalis* 12 days cadmium embryo test assay parameters aquisition characterization.

Position	Development	Malformations	Length - L1	Length - L2	Area	Perimeter	IOD	Hearth beats
Embryo positon in the chorion at the time of measurements: 1 - lateral; 2 - frontal/posterior; 3 - superior/inferior	Developemntal stage (1 - morula; 2 - early trochophore; 3 - mid trochophore; 4 - late trochophore; 5 - velliger; 6 - hippo; 7 - hatched; 8 - dead)	Developemntal abnormalities: 1 - normal; 2 - developmental delay; 3 - edema; 4 - morphological; 5 - swellings; 6 - several; 7 - dead	Longuer axis measurement - length (µm)	Smaller axis measurement – width (µm)	Embryo area (µm²): 48 to 72 h - total embryo; 96 to 168 h - shell area	Embryo perimeter (µm): 48 to 72 h - total embryo; 96 to 168 h - shell perimeter	Distance between eyes (µm): measured in the frontal and superior or inferior positions.	Physiological parameter: hearth beat (beats/minute) counts in the 168 h of exposue.

To ensure robust data analysis, measurements were linked to both the embryo's position and developmental stage, when no significance was observed between the different embryo positions recorded in the same body plan, those were merged. An exploratory approach was favoured in determining the most relevant parameters, considering the known effects of cadmium on development.

In the initial 72 h of incubation, embryos were classified into two positions - lateral and frontal. Beyond this time-point, five positions were recognized (Lateral, Frontal, Posterior, Superior and Inferior). Importantly, no significant measurement distinctions were found between frontal and posterior positions, nor between superior and inferior positions, leading to data consolidation.

During the morula stage (first 24 h), perimeter and area measurements were logged due to the embryo's round shape, which hindered precise position characterization. In the trochophore stages (primarily at 48 and 72 h), measurements included perimeter and embryo area, with position accounting for potential measurement bias. Lateral and frontal/back positions were identified during these stages.

Subsequently, after the fourth day (96 h), when late trochophore and veliger stages predominated, embryos were classified based on chorion position. Measurements were taken across three position groups: lateral, frontal/back, and superior/inferior positions, which were readily distinguishable. These stages marked the point at which adult structures became visible and classifiable, and embryos exhibited distinct movement patterns within the chorion.

Measurements encompassed parameters such as embryo area (total area for morula and trochophore stages, shell area for veliger and hippo stages), perimeter, length (L1), width (L2), area, and perimeter for different positions. Additionally, the interocular distance (IOD) was measured in frontal and superior positions. Finally, heart rate assessments were conducted at 168 h, with both area and perimeter determinations considered in this analysis.

## 4 Limitations

‘Not applicable’.

## Ethics Statement

The authors have read and follow the ethical requirements for publication in Data in Brief and confirming that the current work does not involve human subjects, animal experiments, or any data collected from social media platforms. Further, the carried experiments used invertebrate embryos that are not legislated under the animal experimentation Directives, being one of the main purposes of this methodological proposal to reduce the animal experimentation efforts.

## CRediT authorship contribution statement

**Ricardo Capela:** Investigation, Methodology, Validation, Data curation, Writing – original draft. **Luís Filipe Castro:** Writing – review & editing. **Miguel Machado Santos:** Conceptualization, Writing – review & editing, Supervision. **Jeanne Garric:** Conceptualization, Writing – review & editing, Supervision.

## Data Availability

Lymnaea stagnalis embryo test datafile (Original data) (Mendeley Data)
